# Multifunctional Novel Nanoplatform for Effective Synergistic Chemo-Photodynamic Therapy of Breast Cancer by Enhancing DNA Damage and Disruptions of Its Reparation

**DOI:** 10.3390/molecules28196972

**Published:** 2023-10-07

**Authors:** Zheng Huang, Tong Xian, Xiangyi Meng, Huaisong Hu, Lixia Gao, Jiuhong Huang, Donglin Yang, Kepeng Ou, Bochu Wang, Yimei Zhang

**Affiliations:** 1 National & Local Joint Engineering Research Center of Targeted and Innovative Therapeutics, Chongqing Key Laboratory of Kinase Modulators as Innovative Medicine, Chongqing Collaborative Innovation Center of Targeted and Innovative Therapeutics, College of Pharmacy, Chongqing University of Arts and Sciences, Chongqing 402160, China; zhenghuang@cqwu.edu.cn (Z.H.); xiantong202309@163.com (T.X.); mengxiangyi6120@163.com (X.M.); huhuaisong2023@163.com (H.H.); lixiagao@cqwu.edu.cn (L.G.); huang_jiuhong@163.com (J.H.); dlyang@cqwu.edu.cn (D.Y.); kepeng.ou@outlook.com (K.O.); 2 Key Laboratory of Bio-Theological Science and Technology of Ministry of Education, College of Bioengineering, Chongqing University, Chongqing 400045, China; wangbc2000@126.com

**Keywords:** chemo-photodynamic therapy, DNA damage repair, PARP inhibitor, co-delivery, GSH-responsive

## Abstract

Photodynamic therapy (PDT) is an effective noninvasive therapeutic strategy that has been widely used for anti-tumor therapy by the generation of excessive highly cytotoxic ROS. However, the poor water solubility of the photosensitizer, reactive oxygen species (ROS) depleting by high concentrations of glutathione (GSH) in the tumor microenvironment and the activation of DNA repair pathways to combat the oxidative damage, will significantly limit the therapeutic effect of PDT. Herein, we developed a photosensitizer prodrug (CSP) by conjugating the photosensitizer pyropheophorbide a (PPa) and the DNA-damaging agent Chlorambucil (Cb) with a GSH-responsive disulfide linkage and demonstrated a multifunctional co-delivery nanoplatform (**CSP/Ola** nanoparticles (NPs)) together with DSPE-PEG_2000_ and PARP inhibitor Olaparib (Ola). The **CSP/Ola NPs** features excellent physiological stability, efficient loading capacity, much better cellular uptake behavior and photodynamic performance. Specifically, the nanoplatform could induce elevated intracellular ROS levels upon the in situ generation of ROS during PDT, and decrease ROS consumption by reducing intracellular GSH level. Moreover, the **CSP/Ola NPs** could amplify DNA damage by released Cb and inhibit the activation of Poly(ADP-ribose) polymerase (PARP), promote the upregulation of γ-H2AX, thereby blocking the DNA repair pathway to sensitize tumor cells for PDT. In vitro investigations revealed that **CSP/Ola NPs** showed excellent phototoxicity and the IC_50_ values of **CSP/Ola NPs** against MDA-MB-231 breast cancer cells were as low as 0.05–01 μM after PDT. As a consequence, the co-delivery nanoplatform greatly promotes the tumor cell apoptosis and shows a high antitumor performance with combinational chemotherapy and PDT. Overall, this work provides a potential alternative to improve the therapeutic efficiency of triple negative breast cancer cell (TNBC) treatment by synergistically enhancing DNA damage and disrupting DNA damage repair.

## 1. Introduction

At present, breast cancer (BC) is one of the most commonly malignant tumors that seriously threaten women’s health and lives worldwide because of its high morbidity and mortality rates [1,2,3]. Among the many recently investigated breast cancer treatments, photodynamic therapy (PDT) has attracted great interest and holds great promise since it is a noninvasive, highly accurate, well-controllable and locally effective treatment method [4,5,6]. PDT works through the synergistic action of photosensitizers, excitation light and molecular oxygen. Photosensitizer molecules could be excited under laser irradiation of a specific wavelength when they are internalized by tumor cells, thereby generating a large amount of singlet oxygen (^1^O_2_) and other reactive oxygen species (ROS) [7,8]. The generated ROS could cause rapid oxidative damage to the key biological macromolecules such as DNA, proteins and unsaturated lipids; thus, resulting in damage to key organelles like nucleus and mitochondria and ultimately the death of tumor cells [9,10,11]. Therefore, PDT has come to be considered a desirable non-invasive method for precise treatment of malignant tumors.

However, the therapeutic effects of PDT are still unsatisfactory due to the inherent properties of photosensitizers, the complexity of the tumor microenvironment and existent DNA damage repair mechanisms in tumor cells [12,13,14]. The primary cause of the limited practical application of PDT is the inadequate distribution and insufficient retention of the photosensitizer since the majority of photosensitizers have low water-solubility and high hydrophobicity [15]. Conjugating photosensitizers with hydrophilic fragments (polymers or lipid) to make them nanoparticles or encapsulating them with nanocarriers, whether naturally available or designed and synthesized, may provide an effective way to overcome these difficulties [16,17,18]. Nanocarriers not only can increase the solubility of photosensitizers, but also can provide suitable size and surface characteristics for prolonging blood circulation, which will facilitate the distribution and retention of photosensitizers at the tumor site through the “enhanced permeability and retention (EPR) effect” [19,20,21]. In addition, as a powerful type of ROS scavenger, the high levels of glutathione (GSH) in the tumor microenvironment will substantially deplete ROS generated by excited photosensitizers, decreasing the therapeutic efficiency of PDT. To reduce ROS consumption, developing co-delivery systems of photosensitive therapeutic and GSH-depleting agents or designing GSH-responsive nanocarriers are commonly used strategies [22,23].

Nevertheless, even if the above two obstacles are effectively overcome and ROS-induced DNA damage is achieved, the DNA-damage-associated therapeutic modalities of PDT may not guarantee expectations being fulfilled. The main reason for this is that tumor cells have a complex set of DNA repair mechanisms [24]. During this process, tumor cells immediately activate complex cellular repair machinery to fight DNA damage [25,26,27]. Poly(ADP-ribose) polymerase (PARP), particularly PARP1, is a crucial nuclear enzyme that responds quickly to initiate DNA repair by binding to the single strand damage site of damaged DNA [28,29,30]. It was recently reported that PARP inhibitors have been used to enhance radiotherapeutic, chemotherapeutic and immunotherapeutic agent sensitivity served as a DNA damage repair inhibitor [31,32,33]. Among which, Olaparib (Ola) has been approved by Food and Drug Administration (FDA) for the treatment of recurrent ovarian cancer and advanced breast cancer [34,35]. Moreover, since triple negative breast cancer cells (TNBC) have homologous recombination deficiency that disrupts other DNA repair pathways by synthetic lethality, they are also sensitive to PARP inhibitors [36]. However, studies about PARP inhibition and PDT for TNBC treatment have been rarely reported. Therefore, it is rational to attempt to develop an integrated system based on a photosensitizer and PARP inhibitor for enhanced breast cancer photodynamic therapy.

In addition, the integrated system incorporating a photosensitizer (PS) and PARP inhibitor could be used for a combinational therapy since some therapeutic agent molecules may serve as important components of nanocarriers. It has been demonstrated that monotherapy with a nanomedicine may be ineffective in complete removal of tumors [37]. Combining PDT and chemotherapy has become an attractive strategy for improving the effectiveness of cancer treatment. Of late, the clinical development of PARP inhibitors has evolved from using them as a single drug to the combination of PARP inhibitors with DNA-damaging agents to obtain extra therapeutic benefits from stimulated DNA damage [38,39]. Among these, Chlorambucil (Cb) has become an ideal option as a DNA-damaging agent. Chlorambucil, a member of the nitrogen mustard class of DNA alkylating agents, is one of the first DNA-damaging reagents used for chemotherapy of lymphomas and some solid tumors [40]. The *N, N*-bis(2-chloroethyl)-amine group in the nitrogen mustard of Chlorambucil can rapidly react with nucleic acids, generating the alkylation of genomic DNA and resulting in apoptotic cell death [41]. Therefore, integrating the function of PS, PARP inhibitor and Chlorambucil in a multifunctional nanomedicine will provide an alternative approach that significantly improves the efficacy of synergistic chemo-photodynamic therapy by enhancing DNA damage and dysfunctioning DNA damage repair.

In this work, we constructed a multifunctional nanoplatform to achieve enhanced PDT/chemotherapy synergistic tumor therapy by intelligently integrating the function of pyropheophorbide a (PPa, a photosensitizer), Chlorambucil (Cb, a DNA-damaging drug) and Olaparib (Ola, a PARP inhibitor). In our formulation, the Chlorambucil was coupled with PPa by disulfide linkage to form smartly responsive prodrug (**Cb-SS-PPa**, designated as **CSP**) with dispersion stability. The conjugate **CSP** together with DSPE-PEG_2000_ could self-assemble into nanoparticles (**CSP NPs**), which could further encapsulate Ola to construct co-delivery nanomedicines (**CSP/Ola NPs**). This novel nanoplatform has demonstrated extraordinary potential for enhanced chemo-photodynamic TNBC therapy with unique properties. Firstly, the PEGylation could prolong the circulation of **CSP/Ola NPs** in blood and increase the accumulation in the tumor area by passive targeting [42]. Subsequently, as **CSP/Ola NPs** were internalized by cancer cells, the disulfide bonds of **Cb-SS-PPa** can realize GSH-triggered Cb and Ola release due to the high GSH tumor microenvironment, thereby enhancing DNA damage and dysfunctioning DNA damage repair, respectively. With synergistic action of Cb and Ola, the oxidative damage induced by PPa during PDT progress was further expanded and the enhanced chemo-photodynamic therapy was achieved by promoting cell apoptosis ultimately. Overall, this work provides a potential alternative to improve the therapeutic efficiency of TNBC treatment by synergistically enhancing DNA damage and disrupting DNA damage repair.

## 2. Results and Discussion

### 2.1. Preparation, Characterization and Spectroscopic Properties of **CSP NPs** and **CSP/Ola NPs**

**Scheme 1:**
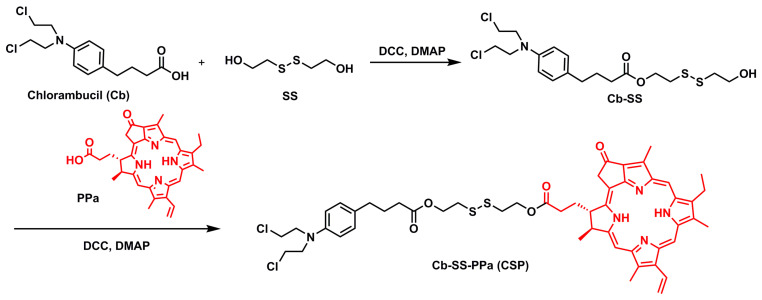
Synthetic routes of target **CSP** prodrug.

The Ola-loaded nanoparticles (**CSP/Ola NPs**) were prepared by encapsulating Ola with a GSH-responsive prodrug, which was coupled with Chlorambucil and PPa by a disulfide linkage (Scheme 1). Briefly, equivalent amounts of Chlorambucil and 2, 2′-dithiodiethanol conjugated to yield disulfide bonds-contained **Cb-SS**, which was further coupled with PPa though ester condensation in one step to obtain the novel photosensitizer-based prodrug (**Cb-SS-PPa**, named **CSP**). The structure of **Cb-SS** and **Cb-SS-PPa** were meticulously confirmed by ^1^H NMR, ^13^C NMR and HRMS (Figures S1–S6).

**Figure 1:**
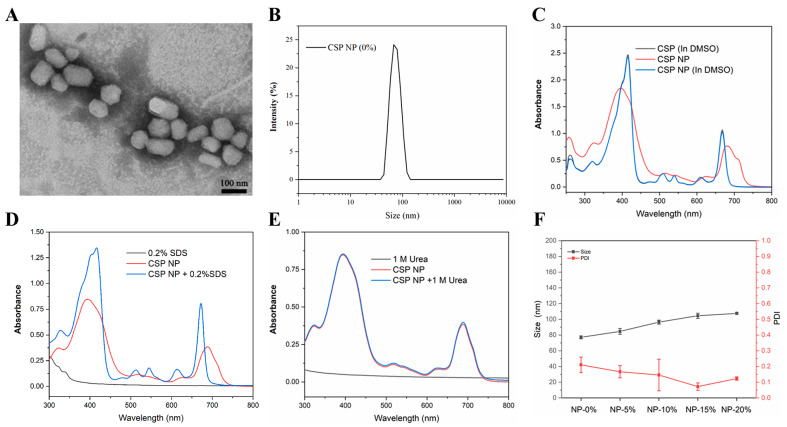
The self-assembly of individual **CSP** to prepare **CSP NPs**. (**A**) The TEM image of **CSP NPs**, scale bar: 100 nm. (**B**) The hydrodynamic size of **CSP NPs**. (**C**) UV–Vis spectra of **CSP** and **CSP NPs** in DMSO or water. (**D**) UV–Vis spectrum of **CSP NPs** in the absence or presence of 0.2% SDS. (**E**) UV–Vis spectrum of **CSP NPs** in the absence or presence of urea. (**F**) The particle size and PDI of various formulations of **CSP NPs** obtained at DSPE-PEG_2000_ content of 0%, 5%, 10%, 15% and 20%, respectively. Data are shown as mean ± SD (*n* = 3).

The excellent self-assembly performances of **CSP** prodrugs are fundamental to realizing further biological applications since the formed nanoparticles can increase the cellular internalization and further co-assemble with other drugs to form multifunctional nanomedicines. The self-assembly behaviors of **CSP** were primarily investigated. The nanoparticles (**CSP NPs**) can easily be formed from **CSP** via the reprecipitation method [43]. As shown in Figure 1A,B, the morphology of the harvested **CSP NPs** was found to be irregularly spherical, observed by transmission electron microscopy (TEM), demonstrating good self-assembly properties. The prepared **CSP NPs** had a hydrodynamic particle size of about 80 nm, measured by dynamic light scattering (DLS).

In order to explore the self-assembly mechanism of the nanoassemblies, the UV–Vis absorption spectrum of **CSP NPs** was recorded under different conditions. Firstly, **CSP NPs** were dissolved in water or DMSO to record the UV–Vis spectrum (Figure 1C). After the formation of nanomedicines, **CSP NPs** had a wider and red-shifted Soret band compared with free **CSP**, proving the existence of noncovalent forces in the self-assembly of **CSP**. However, the UV–Vis spectrum resumed when the **CSP NPs** were dissolved in DMSO. Furthermore, the **CSP NPs** were also treated with hydrophobic sodium dodecyl sulfate (SDS, 0.2% *w*/*v*). As revealed in Figure 1D, the addition of SDS obviously varied the characteristic absorption of **CSP NPs**, demonstrating that hydrophobic interactions should be responsible for the assembly of the nanoassemblies. Specifically, the hydrophobic interaction between the nonpolar groups of Cb and PPa molecules could promote their aggregation and assembly in water solution. Nevertheless, the addition of urea had no effect on the absorption of **CSP NPs** (Figure 1E), suggesting that there was negligible hydrogen-bond interaction between the **CSP**.

The PEG chain was further modified on the **CSP NPs** surface to improve its stability and performance in physiological environments [44]. The various formulations of **CSP NPs** were prepared using the reprecipitation method with 0%, 5%, 10%, 15% and 20% of DSPE-PEG_2000_, respectively. Then, DLS was conducted to characterize the particle size of the obtained **CSP NPs**. After PEGylation, the average particle size of **CSP NPs** slightly increased from ~77 nm to ~107 nm with the increase of DSPE-PEG_2000_ content (Figure 1F). In addition, it was found that **CSP NPs** with 15% DSPE-PEG_2000_ exhibited minimal PDI. Therefore, we will choose this composition ratio to prepare Ola-loaded nanoparticles in the subsequent experiments.

**Figure 2:**
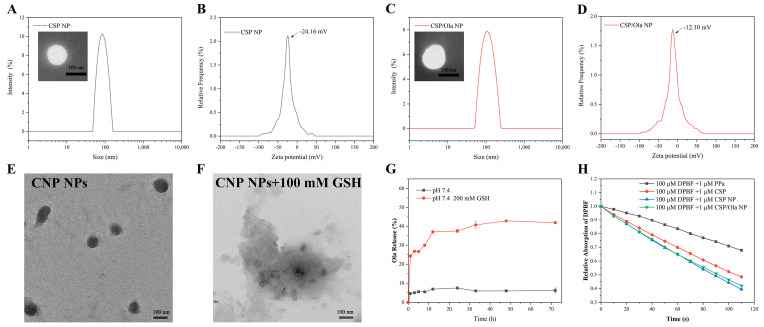
The characterization and spectroscopic properties of PEGylated **CSP NPs** and **CSP/Ola NPs**. (**A**) The hydrodynamic size and TEM image (inserted, scale bar: 100 nm) and the zeta potential (**B**) of PEGylated **CSP NPs**. (**C**) The hydrodynamic size and TEM image (inserted, scale bar: 100 nm) and the zeta potential (**D**) of **CSP/Ola NPs**. (**E**) TEM image of **CSP NPs**, scale bar: 100 nm. (**F**) TEM image of **CSP NPs** after incubation with 100 mM GSH for 48 h. (**G**) The release profiles of Ola from **CSP/Ola NPs** under pH = 7.4 PBS buffer solution in the absence or presence of 200 mM GSH. (**H**) The decay rates of DPBF in PPa, **CSP**, **CSP NPs** and **CSP/Ola NPs** solution under laser irradiation (18 mW/cm^2^). Data are shown as mean ± SD (*n* = 3).

After establishing the optimal DSPE-PEG_2000_ addition content, the PEGylated **CSP NPs** and Ola-loaded nanoparticles (**CSP/Ola NPs**) were prepared using a similar method. Then, DLS and TEM were conducted to characterize the particle size and morphology of the obtained NPs. The results indicated that the particle size of **CSP NPs** was about 105 nm (Figure 2A). After encapsulating Ola, the particle size of **CSP/Ola NPs** slightly increase to 110 nm (Figure 2C). Of special note is that the morphologies of **CSP NPs** and **CSP/Ola NPs** observed by TEM (Figure 2A,C) exhibited a uniform and regular spherical shape after DSPE-PEG_2000_ PEGylation, compared with TEM images obtained by nanoparticles formed from individual **CSP** (Figure 1A), indicating that DSPE-PEG_2000_ can certainly improve the morphological characteristics of nanoparticles. Additionally, as shown in Figure 2B,D, the zeta potential of **CSP NPs** and **CSP/Ola NPs** was found to be −24.16 mV and −12.10 mV, respectively. The negative charge could facilitate the stability of obtained nanoparticles in serum. Furthermore, the particle size of **CSP NPs** and **CSP/Ola NPs** showed no significant difference after 3 weeks (Figure S7A), and exhibited a relatively small fluctuation in 10% fetal bovine serum (FBS), demonstrating good stability and serum tolerance (Figure S7B). This favorable stability of the self-delivery nanomedicine suggested its potential for biomedical applications.

The spectroscopic properties of different formulations were investigated with the UV–Vis spectrometer (Figure S8A,B), in which the PPa shows an absorption peak at 415 nm and 680 nm, respectively. Similar experimental results to the previous Figure 1C were observed mediated by PEGylated **CSP NPs** and **CSP/Ola NPs**. There is a significant shift in the characteristic peaks of **CSP NPs** and **CSP/Ola NPs**. But it was recovered after dissolution into DMSO. Moreover, the loading capacities of PPa, Cb and Ola in **CSP/Ola NPs** were determined to be 32.4% ± 1.5%, 18.4% ± 0.8% and 17.2% ± 1.2%, by using the standard curves measured by UV–Vis spectrometer and high-performance liquid chromatography (HPLC), respectively (Figure S8C,D). The high drug-loading capacity suggested that the co-delivery **CSP/Ola NPs** nanomedicines are capable of encapsulating all of PPa, Cb and Ola efficiently, which will establish a foundation for subsequent biological applications.

The disulfide linkage in the **CSP** prodrug was designed to respond to GSH, therefore, the morphology changes of the **CSP NPs** were observed by TEM to investigate their responsive behavior. As shown in Figure 2E,F, the structure of **CSP NPs** gradually changed from spherical to irregular sheets after incubation with 100 mM GSH for 48 h. The destruction of NPs can be attributed to the dissociation of the disulfide bond by intracellular GSH [45]. After confirming the successful response of the **CSP NPs** to GSH, we further employed a dialysis method to study the in vitro Ola release behavior from **CSP/Ola NPs** in a high concentration GSH (Figure 2G). The in vitro release profile showed that after incubation with 200 mM GSH in PBS (pH 7.4), **CSP/Ola NPs** released ~40% Ola within 48 h, which was significantly higher than the amount released without GSH. These findings confirmed that **CSP/Ola NPs** were stable under physiological conditions and released the cargos in a simulated tumor microenvironment.

Subsequently, DPBF, a singlet oxygen sensor, was utilized to evaluate the ROS generation abilities of various constructions under laser irradiation. It could form an endoperoxide in the presence of ^1^O_2_, thus decreasing DPBF absorption and providing a valuable means of direct monitoring ^1^O_2_ production in vitro. It was found that the absorption of DPBF at 417 nm mediated by free PPa decreased not obviously at the equivalent PPa (Figure S9A). In contrast, when **CSP NPs** or **CSP/Ola NPs** were irradiated, the absorption values decreased dramatically with the increase in irradiation time (Figure S9C,D). As expected, **CSP** experienced a modest change (Figure S9B). The decay rates of DPBF indicated that ^1^O_2_ generation in PPa aqueous solution was much lower than that of self-assembly nanoparticles due to its poor aqueous solubility (Figure 2H). Of special note is that **CSP** showed a certain ^1^O_2_ generation capacity. We speculate that it is due to the self-assembly of part of the **CSP** molecules in aqueous solution. **CSP NPs** showed slightly faster decay rates than **CSP/Ola NPs**, indicating that Ola-loading seemed to have a certain impact on ROS generation ability.

### 2.2. Cell Internalization of **CSP/Ola NPs** for ROS Production, GSH Depletion and PARP Inhibition

**Figure 3:**
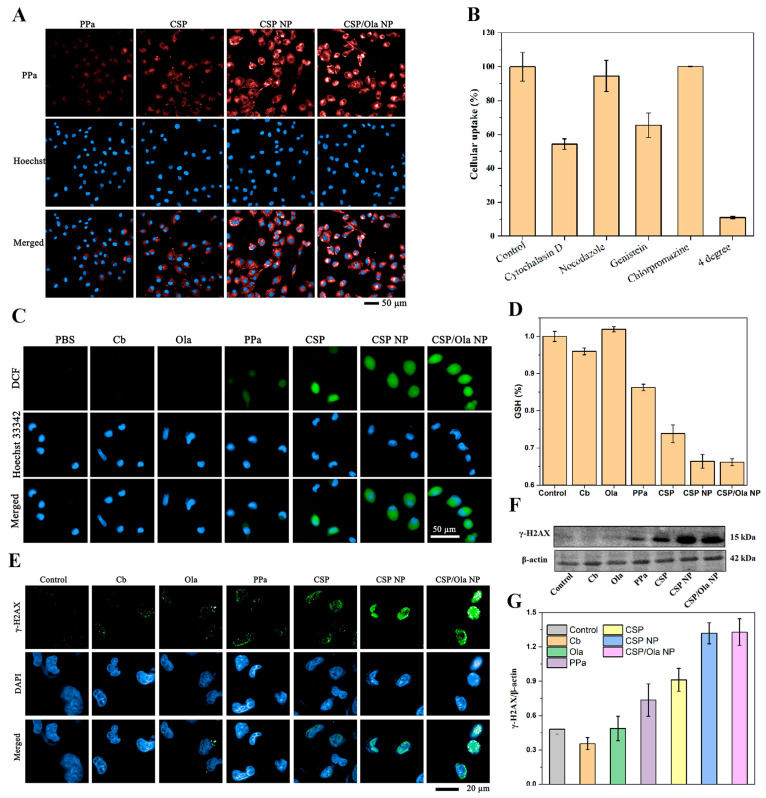
Cell internalization, ROS generation and GSH depletion of **CSP/Ola NPs** to enhance DNA damage by PARP inhibition. (**A**) Images of the MDA-MB-231 cells treated with different formulations at the concentration of 0.2 µM for 4 h captured by the high-content analysis system Operetta CLS™. Scale bar: 50 μm. (**B**) Relative cellular uptake of **CSP/Ola NPs** in MDA-MB-231 cells at 4 °C or in the presence of various endocytic inhibitors quantified by flow cytomety analysis. (**C**) DCF fluorescence intensity of MDA-MB-231 cells incubated with 0.8 µM of Cb, Ola, PPa, **CSP**, **CSP NPs** and **CSP/Ola NPs** under 660 nm laser irradiations with a power of 18 mW/cm^2^ for 2 min captured by the high-content analysis system Operetta CLS™. Scale bar: 50 μm. (**D**) The changes in relative intracellular GSH levels of MDA-MB-231 cells after treatment with different samples for 24 h under 660 nm laser irradiations (18 mW/cm^2^, 2 min). Data are shown as mean ± SD (*n* = 3). (**E**) Confocal microscopic images of the γ-H2AX expression in MDA-MB-231 cells treated with different samples. DAPI staining (blue) and γ-H2AX expression visualized using immunofluorescence (green). Scale bar: 20 μm. (**F**) Western blotting and (**G**) quantitative analysis of γH2AX expressions after various treatments on MDA-MB-231 cells.

Efficient cellular internalization of nanomedicines is a critical factor for therapeutic efficacy. The cellular uptake of prepared nanoplatforms was then studied on model of cell line of MDA-MB-242 231 breast cancer growing in vitro. It was found that the fluorescence signals in the cells mediated by **CSP/Ola NPs** increased gradually with the extension of incubation time and abundant red fluorescent signal could be observed after 4 h (Figure S10A). The fluorescence intensity was approximately unchanged after 8 h (Figure S10B). The results demonstrated the high but not rapid cellular uptake of **CSP/Ola NPs**. We hypothesize that this may be caused by the negative charge on the surface of the **CSP/Ola NPs**. The cellular uptake of the different nanoparticles after 4 h incubation was subsequently compared. As shown in Figure 3A, almost no red fluorescent signal appeared mediated by free PPa because of its poor aqueous solubility. In contrast, a considerable amount of the red fluorescent signal could be observed after treated by **CSP NPs** or **CSP/Ola NPs** nanomedicines, suggesting that the nanostructures prepared by self-assembly could facilitate the cellular uptake. Furthermore, the uptake mechanism of **CSP/Ola NPs** was further investigated. As shown in Figure 3B, the cellular uptake after incubation at 4 °C almost ceased, indicating that **CSP/Ola NPs** enter the cells mainly via an energy-independent pathway. To elucidate the possible cellular uptake pathway of **CSP/Ola NPs**, the cells were treated with different chemical inhibitors including cytochalasin D, nocodazole, genistein or chlorpromazine, which may inhibit the pathway of macropinocytosis, microtubule, caveolae and clathrin-mediated endocytosis, respectively [46]. The results in Figure 3B revealed that cytochalasin D and genistein severally inhibited 46% and 35% of cellular uptake, suggesting that **CSP/Ola NPs** appeared to enter the cell by synergistic micropinocytosis and caveolae-dependent endocytosis.

The intracellular ROS generation ability of different samples was evaluated by detecting the DCF fluorescence intensity of the DCFH-DA fluorescent probe in MDA-MB-231 breast cancer cells since the **CSP/Ola NPs** had revealed high in vitro ^1^O_2_ generation ability (Figure 2H). As shown in Figure 3C, almost no green fluorescence was observed in PBS-, Cb- and Ola-treated cells, suggesting that Cb or Ola alone may not have a therapeutic effect without a photosensitizer. In addition, negligible green fluorescence was found in PPa-mediated cells because of its poor water solubility. By comparison, the cells treated with **CSP NPs** and **CSP/Ola NPs** showed obviously green fluorescence, indicating that a significant quantity of ROS was produced under light irradiation, which is attributed to their nanostructures formed by self-assembly and subsequent high cellular uptake.

We next detected the GSH levels in MDA-MB-231 cells using the GSH and glutathione disulfide (GSSG) assays to investigate the potential capability of the nanoparticles to regulate the cellular GSH levels. It was found that the **CSP NPs** and **CSP/Ola NPs** showed significant GSH inhibition (~35%) compared with the control group (Figure 3D). In contrast, the Cb, Ola and PPa group showed only 5%, 0% and 15% GSH inhibition, respectively. Of special note is that **CSP** prodrug had a moderate GSH inhibition. The remarkable reduced GSH level in the **CSP NPs** and **CSP/Ola NPs** group might be attributed to the stimulus response of disulfide bonds to consume GSH after cell internalization. These results demonstrated that the improved drug delivery efficiency of **CSP NPs** and **CSP/Ola NPs** contributed to the improvement of cell internalization, ROS generation and GSH depletion.

It has been reported that the Ola, as a PARP inhibitor, could suppress DNA damage repair to improve the sensitivity of chemotherapeutic drugs. Generally, the phosphorylated histone γ-H2AX is a biomarker of DNA double bond breakage and its high expression might reveal the enhanced DNA damage and the suppression of DNA damage repair [47]. The expression of γ-H2AX was first assessed by an immunofluorescence assay to verify the effects of **CSP/Ola NPs** on DNA damage and its repair in MDA-MB-231 cells. As shown in Figure 3E, the negligible green immunofluorescence could be observed in free the Cb, Ola, PPa or **CSP** group, indicating that these formulations had a poor ability to damage DNA. It is worth noting that **CSP NPs** induced the appreciable expression of γ-H2AX, implying that the DNA-damaging agent Cb encapsulated in **CSP NPs** functioned. In contrast, the highest expression of γ-H2AX was detected for the **CSP/Ola NPs**, suggesting that **CSP/Ola NPs** presented a synergistic effect on DNA damage and its repair inhibition due to the inherent function of co-loaded Cb and Ola, respectively. The above experimental results were also verified by Western blotting (Figure 3F,G). Collectively, these results confirmed that **CSP/Ola NPs** exhibited enhanced DNA damage and disrupted DNA damage repair capacity.

### 2.3. In Vitro Chemo-Photodynamic Synergistic Antitumor Study

**Figure 4:**
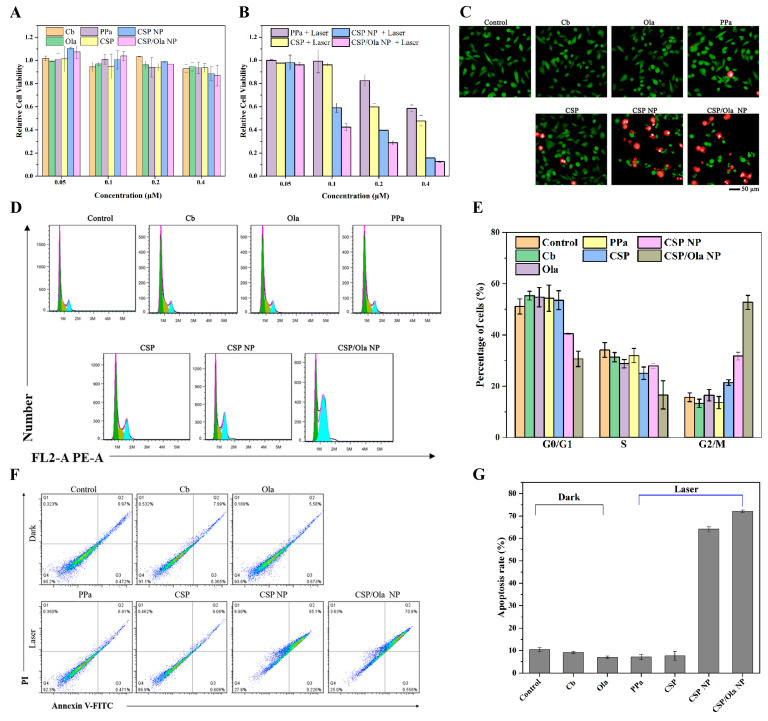
Antitumor effect of **CSP/Ola NPs** in vitro. The MTT assay for detecting the viability of MDA-MB-231 cells after treatment with Cb, Ola, PPa, **CSP**, **CSP NPs** and **CSP/Ola NPs** at different concentrations (**A**) in the absence of light or (**B**) in the presence of 660 nm laser irradiations with a power of 18 mW/cm^2^ for 2 min. (**C**) The live/dead staining of MDA-MB-231 cells treated with Cb, Ola, PPa, **CSP**, **CSP NPs** and **CSP/Ola NPs** under 660 nm laser irradiations (18 mW/cm^2^, 2 min). Live cells were stained with calcein-AM (green) and dead cells were stained with PI (yellow). Scale bar: 50 μm. (**D**) Flow cytometry and (**E**) quantitative analysis of cell cycle distribution in MDA-MB-231 cells after treatment with Cb, Ola, PPa, **CSP**, **CSP NPs** and **CSP/Ola NPs**. (**F**) Annexin V-FITC/PI-based apoptosis and (**G**) quantitative analysis of MDA-MB-231 cells treated with different formulations with or without 660 nm laser irradiations. Data are shown as mean ± SD (*n* = 3).

Encouraged by the excellent cellular internalization, the favorable photodynamic properties and DNA repair capacity, the antitumor effect of **CSP/Ola NPs** was then investigated in vitro. The cytotoxicities of Cb, Ola, PPa, **CSP**, **CSP NPs** and **CSP/Ola NPs** on MDA-MB-231 cells were detected by the MTT assay. Obviously, all the formulations had very weak cytotoxicity in darkness within the tested experimental concentration ranges (0.05–0.4 μM) and the cell viability was more than 90% even with a higher drug concentration (Figure 4A). However, after laser irradiation, **CSP**, **CSP NPs** and **CSP/Ola NPs** showed dose-dependent cytotoxicity due to ROS generation and the cell viability decreased from 95% to 10% (Figure 4B). Even then, the PDT of PPa or **CSP** had a poor therapeutic effect on account of their limited cell internalization. Importantly, **CSP/Ola NPs,** upon light irradiation, inhibited the proliferation of tumor cells more efficiently than that of **CSP NPs**, suggesting a much stronger antitumor capacity than free **CSP NPs**. This was most likely because of the effective delivery of Ola by **CSP/Ola NPs**, which disrupted DNA damage repair by PARP inhibition. The remarkable tumor inhibition effect of **CSP/Ola NPs** was also consistently supported by the cell live/death imaging, which revealed that the **CSP/Ola NPs** group had the largest dead cell population (Figure 4C).

In addition, the impact of **CSP/Ola NPs** on the cell cycle progression of MDA-MB-231 cells was assessed by PI staining and detected by flow cytometry. As shown in Figure 4D,E, the cells in control, free Cb, free Ola, free PPa and **CSP** prodrug groups were mainly in the G0/G1 phase (~55%) and the S phase (~30%), while only ~15% cells were in the G2/M phase. After MDA-MB-231 cells were treated with **CSP/Ola NPs** under laser irradiation, the proportion of cells in the G2/M phase significantly increased to 55.29%, and the percentages of cells in the G0/G1 and S phase were significantly decreased to around 30.69% and 14.02%, respectively. These results suggested that **CSP/Ola NPs** could induce cell cycle arrest in the G2/M phase of mitosis, which led to cancer cell apoptosis.

Furthermore, cell apoptosis analysis was also performed by flow cytometry. As shown in Figure 4F,G, in the absence of light, the percentages of normal cells in Cb and Ola groups were greater than 91%, suggesting a low dark toxicity. However, after laser irradiation, the apoptotic proportion of cells in the **CSP NPs** and **CSP/Ola NPs** groups increased successively. Among which, **CSP/Ola NPs** caused more cell apoptosis than **CSP NPs** (72.0% vs. 64.2%), demonstrating that Ola co-delivered by **CSP/Ola NPs** facilitated the antitumor effect of PDT because Ola-mediated PARP inhibition reduced DNA damage repair and increased the lethality of ROS to tumor cells. Furthermore, **CSP NPs** after light irradiation induced more apoptosis of tumor cells compared to **CSP** prodrug. One of the primary causes should be the increased drug delivery efficiency of agents after assembly into nanomedicine. Additionally, the co-delivery of PPa, Cb and Ola could improve their varied pharmacokinetic profiles, which enhanced the synergistic effect of PDT and DNA repair inhibition. Based on the above, it could be concluded that **CSP/Ola NPs** based co-delivery nanomedicine with combinational chemotherapy and PDT destructed the ROS defensive system and amplified the DNA damage for highly efficient PDT by PARP inhibition.

## 3. Materials and Methods

All chemicals and reagents were obtained commercially and were used as received. Pyropheophorbide a (PPa), Olaparib (Ola) and 1, 3-diphenylisobenzofuran (DPBF) were purchased from Shyuanye Co., Lt (Shanghai, China). 1, 2-distearoyl-sn-glycero-3-phosphoethanolamine-N-[methoxy (polyethylene glycol)-2000] (ammonium salt) (DSPE-PEG_2000_) was purchased from Avanti Polar Lipids (Birmingham, AL, USA). The 2, 2′-dithiodiethanol and Chlorambucil were purchased from Aladdin Co., Lt (Shanghai, China) and Bidepharm Co., Lt (Shanghai, China), respectively. The total glutathione assay kit, 2, 7-dichloroflorescein diacetate (DCFH-DA), Annexin V-FITC apoptosis detection kit and γ-H2AX Immunofluorescence, Calcein/PI cell viability assay kit were obtained from Beyotime Biotechnology (Shanghai, China). The anti-γ-H2AX antibody was obtained from Abcam. The human triple-negative breast cancer cell line (MDA-MB-231 cells) was obtained from the Chinese Academy of Sciences Cell Bank (Shanghai, China).

### 3.1. Synthesis of Cb-SS

Chlorambucil (Cb, 608 mg, 2.0 mmol) was dissolved in anhydrous dichloromethane, and then the 4-Dimethylamino-pyridine (DMAP, 293 mg, 2.4 mmol) and the N, N-dicyclohexyl-carbodiimide (DCC, 495 mg, 2.4 mmol) were added at 0 °C. After 0.5 h, the mixture was added to 2, 2′-dithiodiethanol (**SS**, 616 mg, 4.0 mmol) in anhydrous DCM. The obtained mixture was stirred at room temperature overnight. The reaction solution mixture was cooled and precipitated. After filtering, the filtrate was concentrated by rotary-evaporation. The crude product was purified by silica column chromatography to obtain **Cb-SS** (yield: 67.5%). ^1^H NMR (CDCl_3_, 400 MHz): δ (ppm) = 7.12–7.01 (d, 2H, -CH-), 6.67–6.56 (d, 2H, -CH-), 4.42–4.30 (t, 2H, -CH_2_-), 3.92–3.80 (m, 2H, -CH_2_-), 3.74–3.66 (m, 4H, -CH_2_-), 3.66–3.57 (m, 4H, -CH_2_-), 2.95–2.83 (m, 4H, -CH_2_-), 2.60–2.52 (t, 2H, -CH_2_-), 2.38–2.30 (t, 2H, -CH_2_-), 1.96–1.87 (m, 2H, -CH_2_-). ^13^C-NMR (CDCl_3_, 100 MHz): δ (ppm) = 173.5, 144.4, 130.2, 129.7, 112.3, 62.2, 60.3, 53.6, 41.7, 40.6, 37.1, 34.0, 33.5, 26.7. HRMS (ESI): *m/z* calcd for C_18_H_28_Cl_2_NO_3_S_2_^+^ [M+H]^+^ 440.0882, found 440.0878.

### 3.2. Synthesis of CSP

PPa (50 mg, 0.09 mmol) was dissolved in anhydrous dichloromethane, and then EDCI (100 mg, 0.53 mmol) and DMAP (65 mg, 0.53 mmol) were stirred at 0 °C. After stirring for 0.5 h, Cb-SS (50 mg, 0.11 mmol) was added. The mixture was stirred at room temperature overnight. After concentrated by rotary-evaporation, the crude product was purified by silica column chromatography to obtain **CSP** as a black solid (yield: 82%). ^1^H-NMR (CDCl_3_, 400 MHz): δ (ppm) = 9.50 (s, 1H, -CH-), 9.38 (s, 1H, -CH-), 8.55 (s, 1H, -CH-), 8.05–7.95 (m, 1H, -CH-), 7.12–7.00 (d, 2H, -CH-), 6.66–6.58 (d, 2H, -CH-), 6.32–6.25 (d, 1H, -CH-), 6.20–6.13 (d, 1H, -CH-), 5.31–5.21 (d, 1H, -CH_2_-), 5.15–5.05 (d, 1H, -CH_2_-), 4.53–4.44 (m, 1H, -CH-), 4.39–4.33 (t, 2H, -CH_2_-), 4.32–4.25 (m, 1H, -CH-), 3.97–3.83 (m, 2H, -CH_2_-), 3.72–3.65 (m, 8H, -CH_2_-), 3.64–3.56 (m, 7H, -CH_2_-, -CH_3_), 3.41 (s, 3H, -CH_3_), 3.24 (s, 3H, -CH_3_), 2.79–2.80 (m, 4H, -CH_2_-), 2.60–2.51 (m, 3H, -CH_2_-), 2.38–2.27 (m, 3H, -CH_2_-), 2.05–1.98 (m, 1H, -CH-), 1.96–1.85 (m, 2H, -CH_2_-), 1.84–1.77 (m, 3H, -CH_2_-), 1.73–1.63 (t, 3H, -CH_3_). ^13^C-NMR (CDCl_3_, 100 MHz): δ (ppm) = 196.3, 173.5, 173.4, 171.4, 160.3, 155.2, 150.8, 149.0, 145.0, 144.4, 141.6, 137.9, 136.2, 136.1, 135.9, 131.6, 130.5, 129.7, 129.2, 122.6, 112.3, 106.0, 104.1, 97.2, 93.0, 62.2, 60.3, 53.6, 51.7, 51.7, 50.0, 48.1, 41.7, 40.5, 37.1, 34.0, 33.5, 31.0, 29.9, 26.7, 23.1, 19.5, 17.4, 12.2, 12.1, 11.3. HRMS (ESI): *m/z* calcd for C_51_H_60_Cl_2_N_5_O_5_S_2_^+^ [M+H]^+^ 956.3407, found 956.3419.

### 3.3. Preparation of CSP/Ola NPs

**NPs** was prepared by the self-assembly of **CSP** and DSPE-PEG_2000_ at the weight feed ratio (1:0~20%). Firstly, **CSP** and DSPE-PEG_2000_ were dissolved in DMSO with a concentration of 2 mg/mL. In order to obtain NP-15%, **CSP** (1 mL) and DSPE-PEG_2000_ (335 μL) were mixed and dispersed to water (10 mL). The mixture was stirred for 2 h. Then, the mixture was dialyzed for 3 h to remove DMSO. The solution was filtered (size 220 nm) for removal of free **CSP**. The obtained **NP-15%** was stored under 4 °C for further use. **NPs** in another ratio was prepared by similar methods. The **NP-15%** was also named **CSP NPs**.

**CSP/Ola NPs** was prepared by the self-assembly of **CSP**, **Ola** and DSPE-PEG_2000_. **CSP**, Ola and DSPE-PEG_2000_ were dissolved in DMSO with a concentration of 2 mg/mL. **CSP** (1 mL), Ola (450 μL) and DSPE-PEG_2000_ (335 μL) were mixed and dispersed to water (10 mL). Subsequent preparation was the same as before.

### 3.4. Characterization of CSP/Ola NPs

The diameter distribution and zeta potential were measured by the dynamic light scattering (DLS) Zetasizer nano zsp instrument (Malvern instruments Led). The storage stability was assayed by keeping it at 4 degrees for several days and serum stability was assayed after serum incubation, respectively. The morphology was observed by a JEM-1400 plus system (JEOL, Kyoto, Japan). The drug loading content was determined through UV–Vis spectra and HRMS.

To investigate the assembly mechanism, the UV–Vis spectra of **NP-0%** was measured after incubation in sodium dodecyl sulfate (SDS) or urea solution.

### 3.5. In Vitro ROS Generation

The ROS production was monitored by using DPBF as an ROS indicator. First of all, the aqueous solution (3 mL) of **CSP/Ola NPs** (or free **PPa**, **CSP**, **CSP NPs**) was prepared at a concentration of 1 μM equivalent PPa. Then, 60 μL DPBF DMSO solution (5 mM) was added. The UV–Vis spectra of the obtained mixture was measured. After irradiated by 660 nm laser (18 mW/cm^2^), the UV–Vis spectra of the mixture was obtained every 10 s. The decreased absorption of DPBF at 417 nm was a reflection of the rate of production of ROS.

### 3.6. Cell Culture

Human triple-negative breast cancer cell lines (MDA-MB-231) were cultured in DMEM with 10% FBS and penicillin-streptomycin (100 mg/L). MDA-MB-231 cells were kept in a humidified incubator with an atmosphere of 5% CO_2_ at 37 °C.

### 3.7. Cellular Uptake and Endocytic Pathway

MDA-MB-231 cells (1 × 10^4^ cells/well) were seeded and cultured in 96-well plates. Cells were, respectively, treated with free **PPa**, **CSP**, **CSP NPs** and **CSP/Ola NPs** (0.2 μM equivalent PPa) for 4 h. Then, the nuclei were stained by Hoechst 33342 (λ_ex_ = 346 nm, λ_em_ = 460 nm) for 10 min. After the cells were washed, the intracellular fluorescence was observed using the high content analysis system.

To evaluate the cellular uptake in a time-dependent manner, MDA-MB-231 cells (1 × 10^4^ cells/well) were seeded and cultured in 96-well plates. The cells were treated with **CSP/Ola NPs** (0.2 μM equivalent PPa). At timed intervals, the intracellular fluorescence was observed using the high content analysis system.

To evaluate the endocytic pathway, common endocytosis inhibitors were used. MDA-MB-231 cells (1 × 10^5^ cells/well) were seeded and cultured in 6-well plates. Cells were pretreated with chlorpromazine (31 µM), genistein (275 µM), nocodazole (16 µM), and cytochalasin D (2 µM) for 0.5 h, respectively. Another experimental plate was pretreated at 4 °C for 0.5 h. **CSP/Ola NPs** (0.2 µM) was added and incubated with the inhibitor or at 4 °C. After 4 h, the cells were washed and collected. The fluorescence intensity was determined by Accuri C6 flow cytometer (BD, San Jose, CA, USA). Untreated cells were used as a control.

### 3.8. Intracellular ROS Generation

MDA-MB-231 cells were seeded and cultured in 96-well plates. The cells were treated with different formations (0.8 μM equivalent PPa) for 24 h. The cells were stained with DCFH-DA for 30 min. Then, the cells were washed and irradiated by a 660 nm laser (18 mW/cm^2^) for 2 min. The intracellular fluorescence of DCF was observed using the high content analysis system (λ_ex_ = 488 nm, λ_em_ = 525 nm).

### 3.9. Intracellular Glutathione (GSH) Levels

MDA-MB-231 cells were seeded and cultured in 6 cm dishes. The cells were treated with different formations (1.2 μM). After 24 h of incubation, the cells were collected and assayed utilizing the Total Glutathione Assay Kit (Beyotime Biotechnology, Shanghai, China).

### 3.10. MTT Assays

MDA-MB-231 cells were seeded and cultured in 96-well plates. The cells were treated with different formations with gradient concentration. After 24 h, the culture medium was replaced by fresh complete medium. For the light group, the cell was irradiated by a 660 nm laser (18 mW/cm^2^) for 2 min. Then, the cells were further incubated for 24 h. Afterwards, each well was added with 20 μL MTT solution (5 mg/mL) and incubated for 4 h. the cell media was replaced by DMSO (200 μL). Cell viability was assessed by absorbance at 570 nm with the microplate reader (Bio-Tek, Winooski, VT, USA).

### 3.11. Cell Apoptosis Detection

MDA-MB-231 cells (1 × 10^6^ cells/well) were seeded and cultured in 6 cm dishes. The cells were treated with different formulations (0.2 μM). After 24 h, the culture medium was replaced by fresh complete medium. For the light group, the cells were irradiated by a 660 nm laser (18 mW/cm^2^) for 2 min. The cells were further incubated for 24 h and were assayed by the Annexin V-FITC Apoptosis Detection Kit (Beyotime Biotechnology, Shanghai, China).

### 3.12. Live and Dead Cell Staining

MDA-MB-231 cells were seeded and cultured in 96-well plates. The cells were treated with different formulations (0.2 μM). After 24 h incubation, the culture medium was replaced by fresh complete medium. The cells were irradiated by a 660 nm laser (18 mW/cm^2^) for 2 min and further incubated for another 24 h. The cells were stained with calcein-AM and PI for 30 min. The intracellular fluorescence was observed using the high content analysis system.

### 3.13. γ-H2AX Immunofluorescence

MDA-MB-231 cells were seeded and cultured in 96-well plates. The cells were treated with different formulations (0.2 μM). After 24 h, the culture medium was replaced by fresh complete medium. The cells were irradiated by a 660 nm laser (18 mW/cm^2^) for 2 min and further incubated for another 4 h. Then, the cells were assayed utilizing the DNA damage assay kit by γ-H2AX Immunofluorescence (Beyotime Biotechnology, Shanghai, China).

### 3.14. Western Blotting

MDA-MB-231 cells were seeded and cultured in 6 cm dishes. The cells were treated with different formulations (0.2 μM). After 24 h, the culture medium was replaced by fresh complete medium. The cells were irradiated by a 660 nm laser (18 mW/cm^2^) for 2 min or in darkness and further incubated for another 4 h. Then, the cells were treated with RIPA lysis buffer. The extracted proteins were used for Western blotting analysis.

## 4. Conclusions

In summary, we have designed and synthesized a novel prodrug by conjugating the photosensitizer PPa and DNA damaging agent Cb with a GSH-responsive disulfide linkage. The multifunctional **CSP/Ola NPs** co-delivery nanomedicine was prepared through the self-assembly of the photosensitizer prodrug, DSPE-PEG_2000_ and the PARP inhibitor Ola to enhance oxidative damage for highly efficient tumor inhibition. The **CSP/Ola NPs** features excellent physiological stability, efficient loading capacity, much improved cellular uptake behavior and photodynamic performance. In addition, The **CSP/Ola NPs** could efficiently deliver the therapeutic agents to tumor cells and release them in the tumor microenvironment in a GSH-responsive manner. Moreover, the nanoplatform could induce elevated intracellular ROS levels upon the in situ generation of ROS during PDT and decrease ROS consumption by reducing the intracellular GSH level, which was attributed to the stimulus response reaction of the disulfide bond after cell internalization. More importantly, **CSP/Ola NPs** could amplify DNA damage by released Cb and prevent DNA damage repair by inhibiting the activity of PARP, thereby hindering the ROS defense system and sensitizing tumor cells to ROS. In vitro investigations revealed that **CSP/Ola NPs** showed excellent phototoxicity and promoted cell apoptosis by amplifying the DNA damage, leading to an effective MDA-MB-231 breast cancer cell suppression. The IC_50_ values of **CSP/Ola NPs** was as low as 0.05–01 μM after PDT. In particular, the synergetic effect of combination therapy presented the best anticancer efficacy in vitro as compared with chemotherapy/PDT alone. Overall, this rationally designed **CSP/Ola NPs** with combinational chemotherapy and PDT in this work improved the PDT efficacy by sensitizing tumor cells to ROS and providing a possibility for combinatorial cancer therapy of tumors simultaneously.

## Data Availability

The data are contained in the article.

## Supplementary Materials

The following supporting information can be downloaded at: https://www.mdpi.com/article/10.3390/molecules28196972/s1, Figure S1: The ^1^H NMR spectra of **Cb-SS**; Figure S2: The ^13^C NMR spectra of **Cb-SS**; Figure S3: The HRMS spectra of **Cb-SS**; Figure S4: The ^1^H NMR spectra of **Cb-SS-PPa**; Figure S5: The ^13^C NMR spectra of **Cb-SS-PPa**; Figure S6: The HRMS spectra of **Cb-SS-PPa**; Figure S7: The stability properties of **CSP NPs** and **CSP/Ola NPs**. (A) Mean particle sizes of **CSP NPs** and **CSP/Ola NPs** at different time points. (B) Mean particle sizes of **CSP NPs** and **CSP/Ola NPs** in the presence of PBS and 10% FBS; Figure S8: (A) UV–Vis spectra of PPa, Cb and **CSP** in DMSO, **CSP NPs** aqueous solution. (B) UV–Vis spectra of Ola, **CSP**, **CSP/Ola NPs** in DMSO, **CSP NPs** aqueous solution. (C) The standard curves of PPa measured by HPLC. (D) The standard curves of Ola measured by HPLC; Figure S9: UV–Vis spectra of 100 μM DPBF in 1 μM PPa (A), 1 μM **CSP** (B), 1 μM **CSP NPs** (C) and 1 μM **CSP/Ola NPs** (D) solution before and after 660 nm laser irradiations for different times; Figure S10: The in vitro cellular uptake ability of **CSP/Ola NPs** at the concentration of 0.2 µM for different times. The images (A) and the relative fluorescence intensity (B) of PPa were studied by the high content analysis system operetta CLS^TM^.
