# Supplementary material for: Multifunctional Novel Nanoplatform for Effective Synergistic Chemo-Photodynamic Therapy of Breast Cancer by Enhancing DNA Damage and Disruptions of Its Reparation
Source: Molecules. 2023 Oct 7;28(19):6972. doi: 10.3390/molecules28196972 (PMC10574765; doi:10.3390/molecules28196972)
Supplement: Supplementary file 1 [file molecules-28-06972-s001.zip › molecules-2638818-supplementary.pdf]

Supplementary Material

Multifunctional Novel Nanoplatform for Effective Synergistic Chemo-Photodynamic Therapy of Breast Cancer by Enhancing DNA Damages and Disruptions of their Reparation

Zheng Huang [1,2], Tong Xian [1], Xiangyi Meng [1], Huaisong Hu [1], Lixia Gao [1], Jiuhong Huang [1], Donglin Yang [1], Kepeng Ou [1], Bochu Wang [2] and Yimei Zhang [1,*]

[1] National & Local Joint Engineering Research Center of Targeted and Innovative Therapeutics, Chongqing Key Laboratory of Kinase Modulators as Innovative Medicine, Chongqing Collaborative Innovation Center of Targeted and Innovative Therapeutics; College of Pharmacy, Chongqing University of Arts and Sciences, Chongqing, 402160, China; zhenghuang@cqwu.edu.cn (Z.H.); 1294007349@qq.com (T.X.); 1971751541@qq.com (X.M.); 3325710019@qq.com (H.H.); LixiaGao@cqwu.edu.cn (L.G.); huang_jiuhong@163.com (J.H.); dlyang@cqwu.edu.cn (D.Y.); kepeng.ou@outlook.com (K.O.)

[2] Key Laboratory of Bio-theological Science and Technology of Ministry of Education, College of Bioengineer ing, Chongqing University, Chongqing 400045, China; wangbc2000@126.com

* Correspondence: yimeizhang@cqwu.edu.cn; Tel.: +86–023-6116–2783

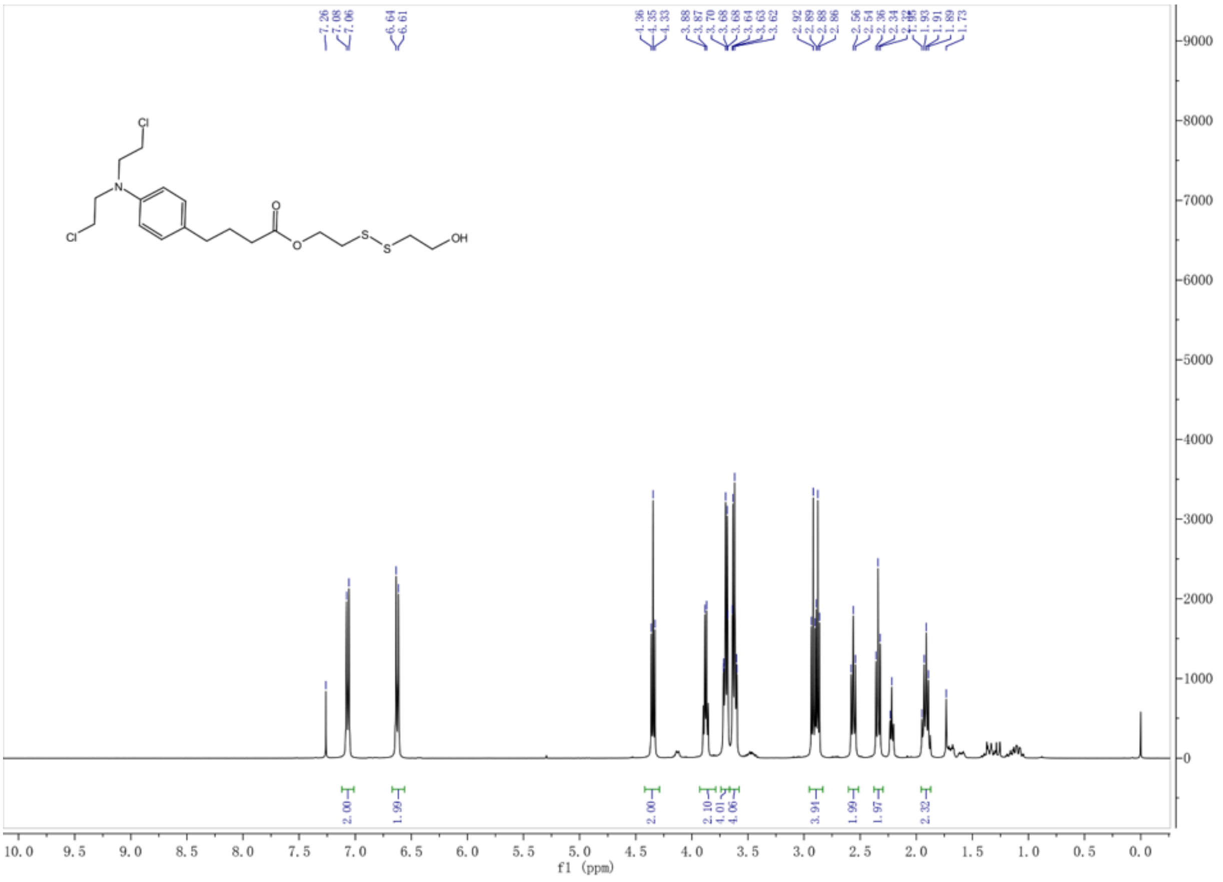

Figure S1. The ^{1}H NMR spectra of **Cb-SS**.

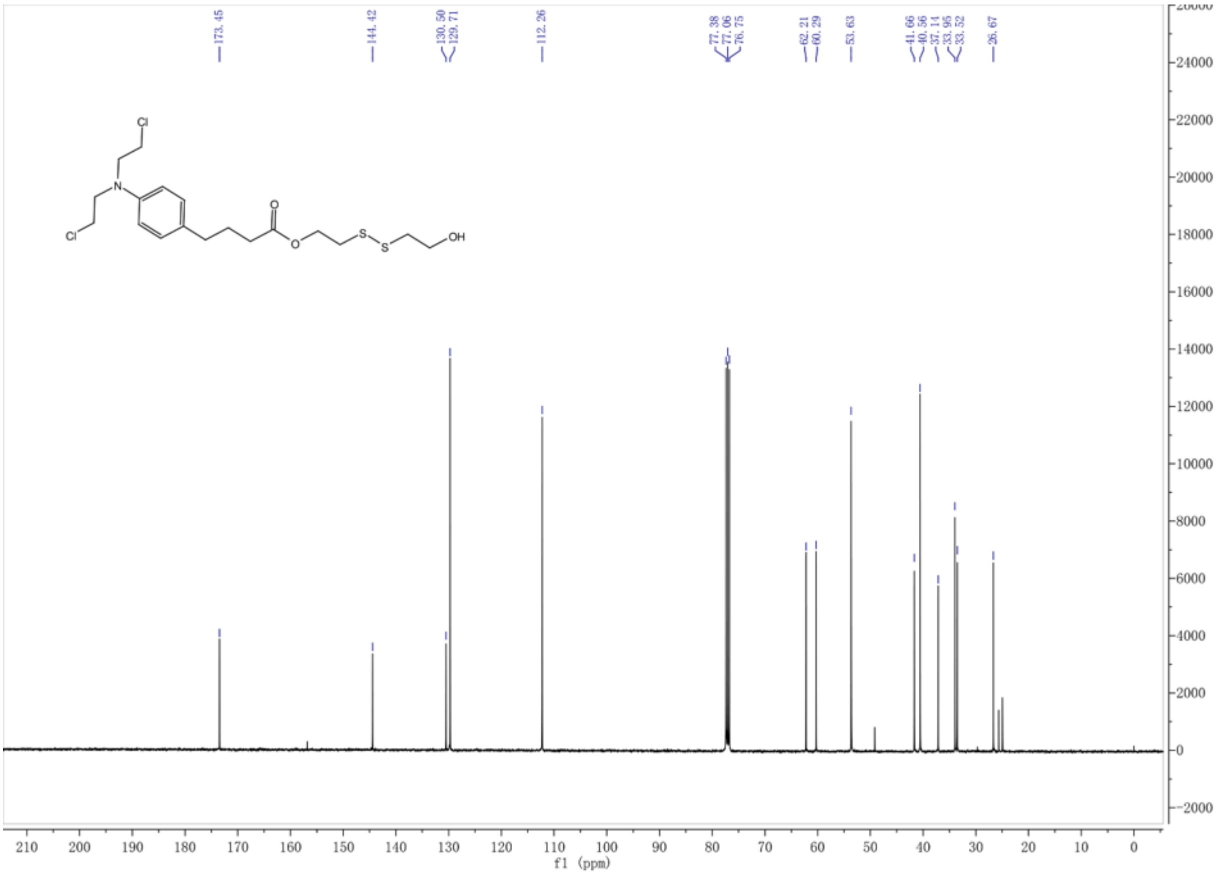

Figure S2. The ^{13}C NMR spectra of **Cb-SS**.

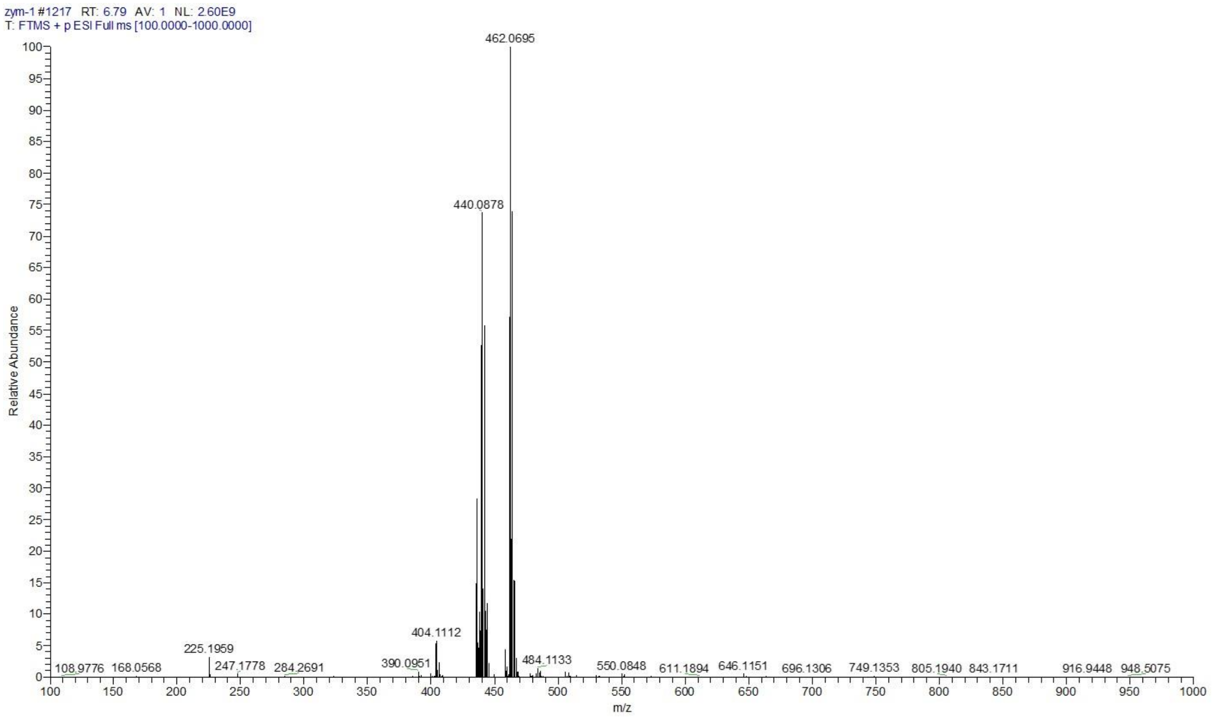

Figure S3. The HRMS spectra of **Cb-SS**.

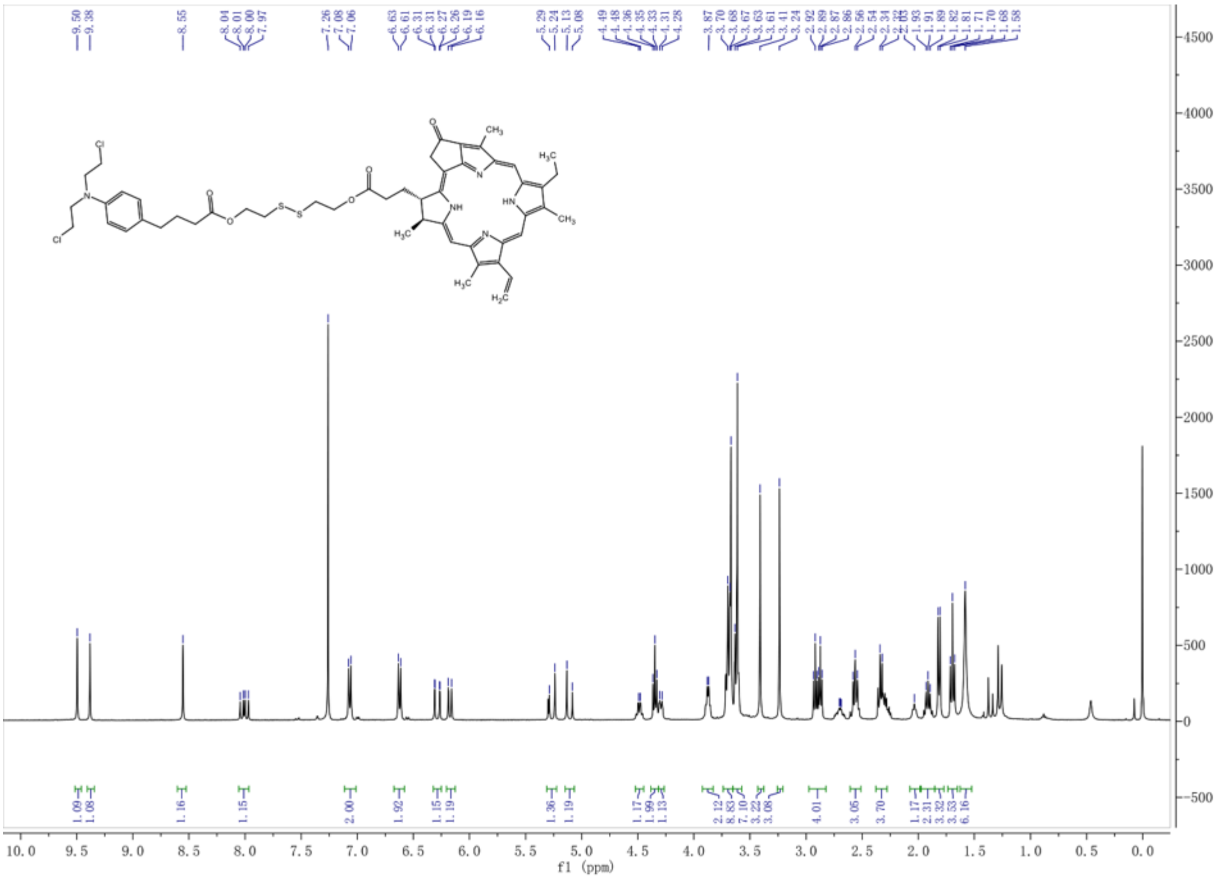

Figure S4. The ^{1}H NMR spectra of **Cb-SS-PPa**.

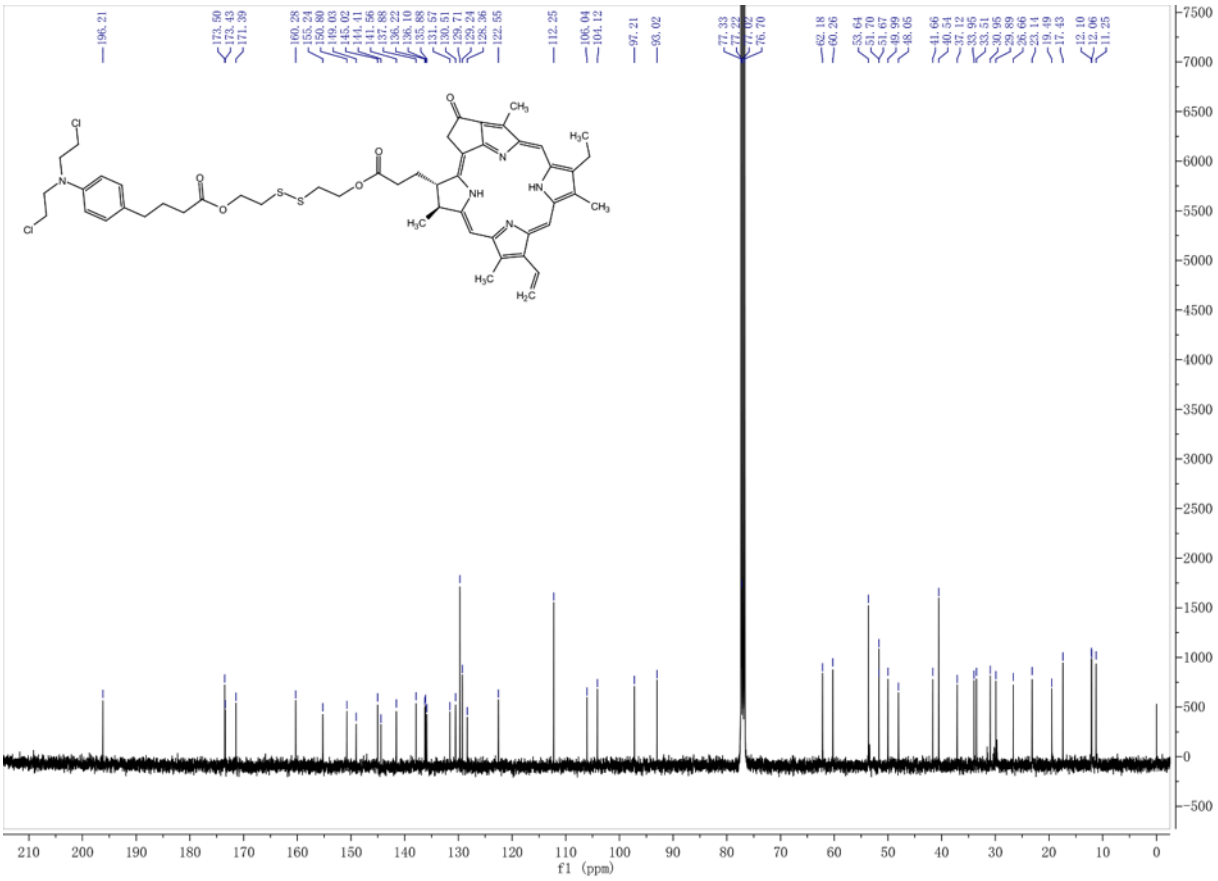

Figure S5. The ^{13}C NMR spectra of **Cb-SS-PPa**.

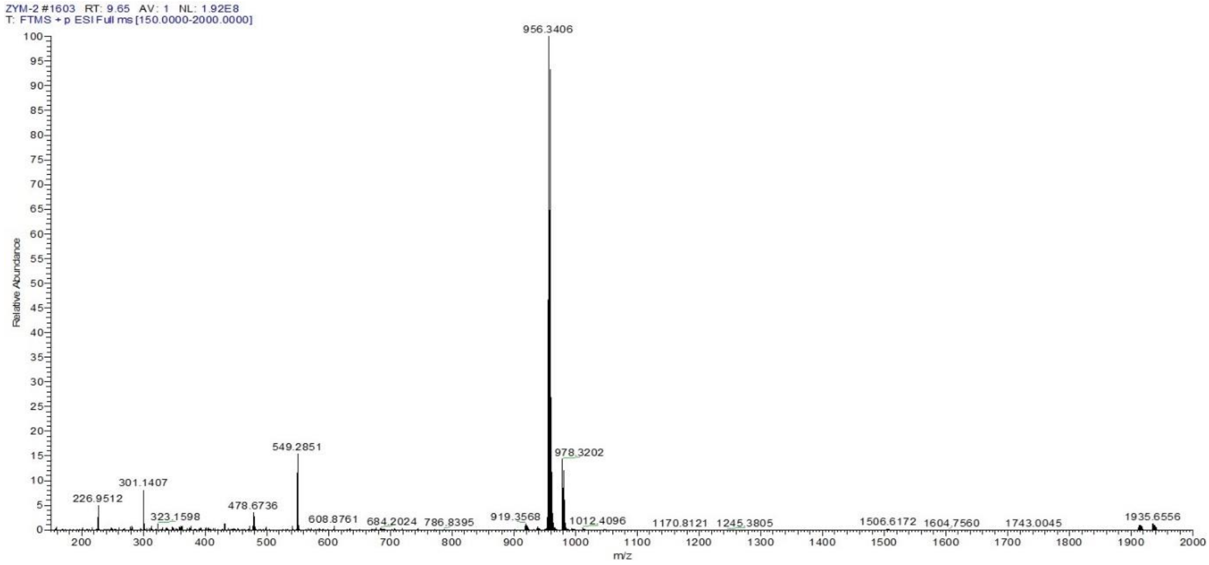

Figure S6. The HRMS spectra of **Cb-SS-PPa**.

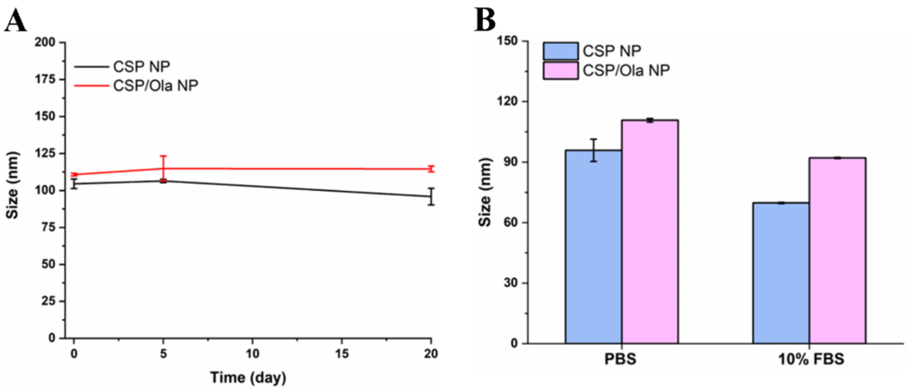

Figure S7. The stability properties of **CSP NPs** and **CSP/Ola NPs**. (A) Mean particle sizes of **CSP NPs** and **CSP/Ola NPs** at different time points. (B) Mean particle sizes of **CSP NPs** and **CSP/Ola NPs** in the presence of PBS and 10% FBS.

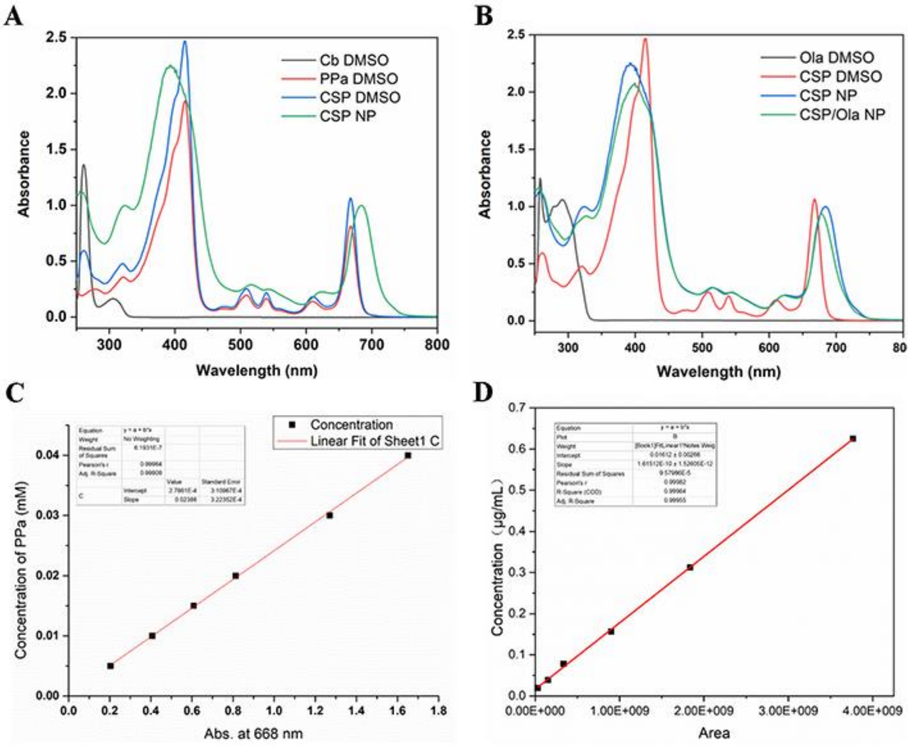

Figure S8. (A) UV–vis spectra of PPa, Cb and **CSP** in DMSO, **CSP NPs** aqueous solution. (B) UV–vis spectra of Ola, **CSP**, **CSP/Ola NPs** in DMSO, **CSP NPs** aqueous solution. (C) The standard curves of PPa measured by HPLC. (D) The standard curves of Ola measured by HPLC.

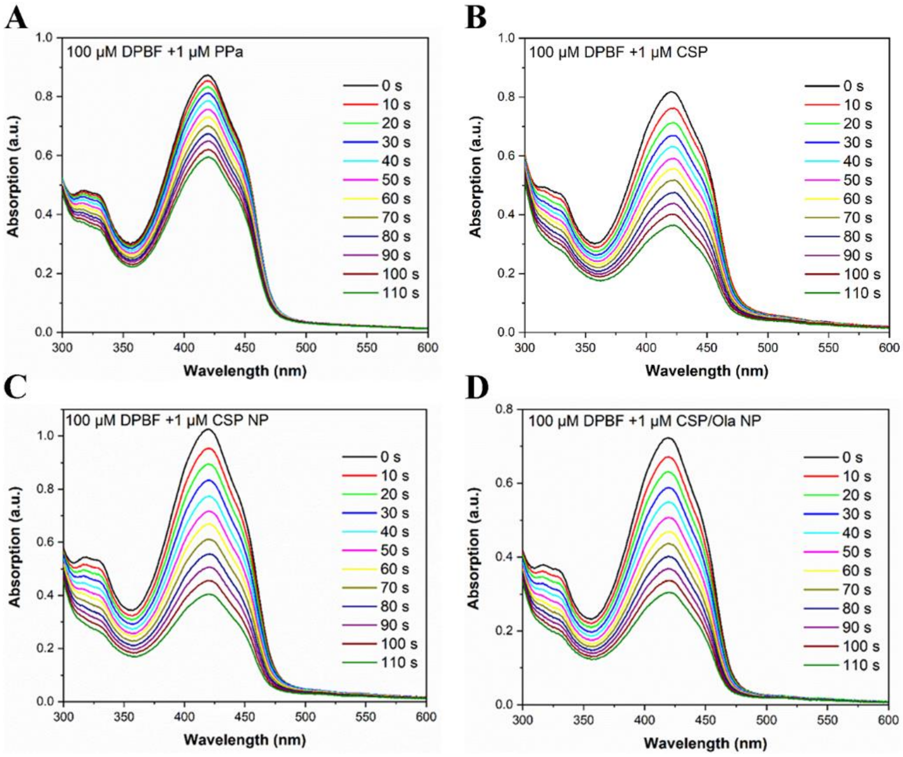

Figure S9. UV–vis spectra of 100 μM DPBF in 1 μM PPa (A), 1 μM **CSP** (B), 1 μM **CSP NPs** (C) and 1 μM **CSP/Ola NPs** (D) solution before and after 660 nm laser irradiations for different time.

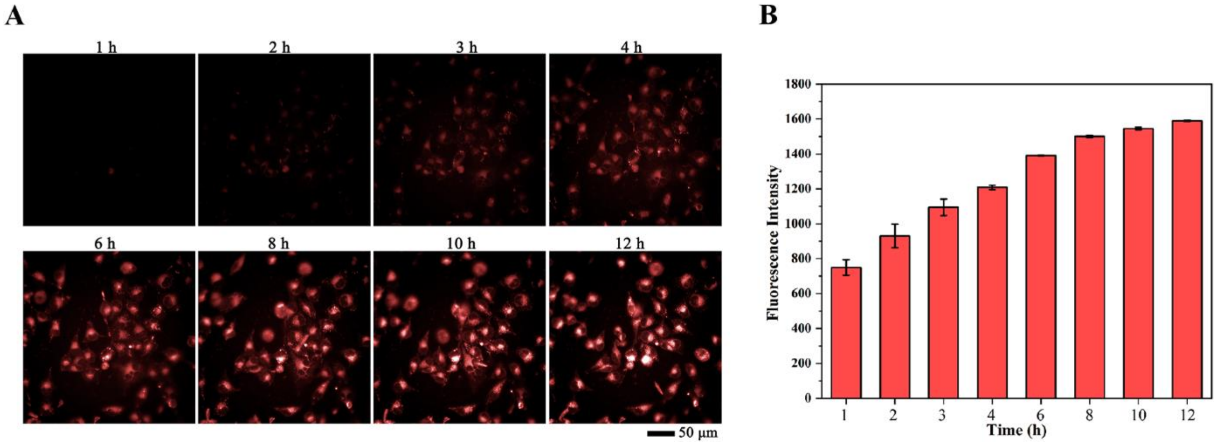

Figure S10. In vitro cellular uptake ability of **CSP/Ola NPs** at the concentration of 0.2 μM for different times. The images (A) and the relative fluorescence intensity (B) of PPa were studied by high content analysis system-operetta CLS™.
